# Nutritional status, hemoglobin level and their associations with soil-transmitted helminth infections between Negritos (indigenous) from the inland jungle village and resettlement at town peripheries

**DOI:** 10.1371/journal.pone.0245377

**Published:** 2021-01-13

**Authors:** Azdayanti Muslim, Yvonne Ai-Lian Lim, Sakinah Mohd Sofian, Syahrul Azlin Shaari, Zaini Mohd Zain

**Affiliations:** 1 Faculty of Medicine, Department of Parasitology, University of Malaya, Kuala Lumpur, Malaysia; 2 Faculty of Medicine, Department of Medical Microbiology and Parasitology, Universiti Teknologi MARA, Sungai Buloh, Selangor, Malaysia; 3 Centre for Malaysian Indigenous Studies (CMIS), University of Malaya, Kuala Lumpur, Malaysia; 4 Centre of Excellence for Research in AIDS (CERiA), University of Malaya, Kuala Lumpur, Malaysia; 5 Faculty of Medicine, Institute of Medical Molecular Biotechnology (IMMB), Universiti Teknologi MARA, Sungai Buloh, Selangor, Malaysia; UCSI University, MALAYSIA

## Abstract

This study compared the current nutritional status, hemoglobin levels and their associations with soil-transmitted helminth (STH) infections between two categories of Negritos (indigenous): (i) Inland Jungle Villages (IJV) (ii) and Resettlement Plan Scheme (RPS) near town peripheries, decades after redevelopment and demarginalization. A total of 416 Negritos (IJV: 149; RPS: 267) was included for nutritional profiling based on anthropometric analysis. However, only 196 (IJV: 64; RPS: 132) individuals consented to blood taking for the hemoglobin (Hb) measurements. Subsequently, the association of undernutrition and anemia with STH infections were determined based on univariate and multivariate logistic regression analyses. The overall prevalence of stunting, wasting, and underweight amongst children and adolescents (n = 343) were 45.8%, 42.3% and 59.1%, respectively. In adults (n = 73), the prevalence of underweight was low (6.8%) but overweight and obese was prominent (26.0%). For anemia (n = 196), an overall prevalence rate of 68.4% were observed with 80% and 70.4% of children aged 2–6 y/o and aged 7–12 y/o, respectively being anemic. Comparatively, the prevalence of underweight (WAZ) was significantly higher in the RPS versus the IJV (P = 0.03) In the IJV, children aged ≤ 6 y/o and having STH poly-parasitism were associated with underweight (P = 0.01) and moderate-severe *T*. *trichiura* infection was associated with anemia. Whilst in the RPS, underweight was highly associated with only *T*. *trichiura* infection (P = 0.04). Wasting was significantly associated with young children aged ≤10 in both IJV (P = 0.004) and RPS (P = 0.02). Despite efforts in improving provision of facilities and amenities among the indigenous, this study highlighted a high magnitude of nutritional issues among the Negritos especially those in the RPS and their likely association with STH infections and decades of demarginalization. Joint nutritional intervention strategies with mass anti-helminthic treatment are imperative and urgently needed to reduce the undernutrition problems especially among indigenous children.

## Introduction

Diet and good nutritional status are important determinants for maintaining optimum health in humans. Any deficiencies or imbalance in nutrient intakes may cause mortality risk, such as stunting, wasting and underweight especially among children. It was estimated that child malnutrition has contributed to 54% of death among children in developing countries [1,2]. Meanwhile, nutritional excesses (overweight and obesity), particularly among the adult population could lead or exacerbate various non-communicable diseases such as diabetes and cardiovascular problems in later life course [3].

In Malaysia, the undernutrition problems are more pronounced among the indigenous Orang Asli (OA) (i.e. the Proto Malay, Senoi and Negrito tribes) as compared to their non-indigenous counterparts. The prevalence of childhood undernutrition among the OA has been observed to be 1–3 times higher (underweight: 29.2%–50.9%; stunting: 28.0%–76.2%; wasting: 12.5%–30.0%) [4–7] than national averages (13.7%–20.7%) [8]. Anemia [9] and protein-energy malnutrition [10] are also highly prevalent. These burdens are often reported to be in a vicious cycle with the soil-transmitted helminth (STH) infections, in which one predisposes the other [2,11]. Although inconsistent, this relationship has been supported in recent studies, which suggested severe STH infections as risk factors for malnutrition especially among OA children [10,12,13]. This knowledge was supported by current statistics that reported high OA child mortality rate (51.7 versus 8.1/1000 live births for national averages) [14] which was mainly caused by diarrhoea, a symptom which can severely worsen in both undernourished and STH-infected children [15,16].

Interestingly, these malnutrition problems were uncommon in OA during the pre-resettlement era (before the 1978s) [17–19] despite highly plagued with STH infections. Evidences from studies undertaken during that time showed that the nutrition of OA children was adequate [20,21] and similar to urban sample [22]. Apart from that, the serum protein profiles [17] and blood pressure were reported to be healthy and normal [23] with little risk of coronary heart disease observed amongst the OA [24]. Therefore, the OA’s health was considered satisfactory in those days, sustained by their active nomadic lifestyle and ample jungle food resources which provided adequate nutrient [19].

The more recent scenario described earlier certainly deferred from the primary aims of OA resettlement as part of redevelopment and demarginalization programs which were to increase the quality of health and wellbeing. It should be noted that, this redevelopment has been initiated since approximately 42 years ago (in 1978). As a consequence, the OA have been exposed to various environmental-cultural and socio-economic changes. Some studies have reported the positive effects of demarginalization efforts, however other studies showed otherwise [18,19,25]. In our previous findings, we have highlighted problems related with higher burden of STH infections among OA who lived in resettlement closer to town or mainstream societies as compared to those in the inland jungle territories [26].

As a sequel, this present study aimed to compare the current status of malnutrition, anaemia as well as their associations with STH infections and other selected variables between the two categories of Negritos (indigenous): (i) those who are still living within inland forest territories (referred as IJV) and (ii) those living in resettlement near town peripheries (referred as RPS) after decades of post-resettlement. The Negrito tribe was chosen because of the scarcity of available information on the status of malnutrition amongst this tribe and their unique characteristics as avid hunter gatherer in the past, hence presumed to undergo the most significant changes during the transition towards modernization [27]. In view of the differing degree of development in both IJV and RPS Negritos, we postulate some variances in malnutrition status and pattern of association with STH due to differences in environmental-cultural settings. These findings are important in providing evidence-based data for the formulation of effective customized strategies for health improvement with regards to malnutrition and their association with STH infections, as some of the Negrito subtribes are now facing the danger of extinction due to their extremely small population size.

## Materials and methods

### Ethical approval and consent

The study was approved by the Ethics Committee of the Universiti Teknologi MARA [reference no: 600- IRMI (5/1/6)] and National Medical Research Register (NMRR), Ministry of Health, Malaysia [(NMRR-17-3055-37252 (IIR)]. Further permission to conduct the study among the OA Negritos was obtained from the Department of Orang Asli Development (Jabatan Kemajuan Orang Asli or JAKOA) [Reference no: JAKOA/pp.30.052J1d9 (29)].

A customary visit to the head of OA was carried out at the selected villages prior to sample collection. During the sampling session, a short briefing on the purpose and study procedures were briefly informed to prospective Negrito participants. Those who agreed to participate were asked to complete a written informed consent, either signed or thumb-printed, witnessed by the accompanying JAKOA officer (s). For participants < 12 y/o, permission and consent were obtained from their parents/ legal guardian. All participants were informed on their right to withdraw from the study at any time without prior notice and prejudice. At the end of the study, the participants were treated with anthelminthic treatment as well as received some contributions in terms of staple foods (e.g. rice, milk, flour, eggs), slippers, clothes and hygiene kits (e.g. soup, tooth brush and toothpaste).

### Study design, sample size and sample collection

In this cross-sectional study, a similar cohort of contemporary Negrito hunter gatherers as described comprehensively in a previous study on STH infection [26], were further examined for nutritional status based on anthropometric indices and anemia status. In brief, the Negritos were divided into two categories based on locality and lifestyle: (i) Inland Jungle Villages, IJV (the community whose villages are located interiorly in the low land forest, undergoing *in situ* development and managed to retain some of their traditional lifestyles), and (ii) Resettlement Plan Scheme, RPS (the community which have been regrouped and relocated to new resettlement areas at the RPS areas or near towns, closer to the mainstream communities, undergoing *ex situ* development and presumed to be relatively more advanced). The study design, summary of sample collection and community categories are shown in **S1 Fig**.

The sample size was calculated using PS Power and Sample Size Calculation Software based on two proportions (prospective study) according to the formula provided by Wang & Chow [28]. By taking 5% significance level (α), 80% power of study, the prevalence of significant underweight (to represent malnutrition problems) in RPS was 56.5% [10] and assuming prevalence of 30.0% among the IJV Negritos, the minimum sample size needed was 112 (56 from each community). By adjusting the 15% attrition rate, the minimum sample size required was 128.

As described in previous study [26], a total of 430 Negritos was first recruited. However subsequently only 416 Negritos [IJV: 149 (38.5%); RPS: 267 (64.2%)] with a complete paired stool samples and anthropometric data with age ranging from 2–64 years (median: 10 years) were included. Given the wide age range, the participants from each IJV and RPS were divided into two groups; (i) children and adolescents (age 2–19 years old or ≤ 19 years), and (ii) adults (age 20–64 years old or > 19 years). Briefly, fecal samples were examined microscopically for the prevalence of STH infections (*Trichuris trichiura*, *Ascaris lumbricoides* and hookworm) using the iodine wet mount, formalin-ether sedimentation, modified Trichrome staining and Ziehl-Neelsen staining methods. Kato-Katz techniques was performed to determine the STH intensity (worm burden) for each STH species.

For calculation of anthropometric indices among children and adolescents, the age was further divided into four age groups (years); 2–5; 6–8; 9–10; 11–19 in order to determine the most affected age-groups with undernutrition problems. In addition, these particular age ranges were subscribed to provide equal or similar number of participants for each age group, for example those aged from 15–19 y/o (n = 24) were combined with 11–14 y/o group (n = 76) due to extremely low number of participants. For hemoglobin (Hb) examination, only 196 Negritos [IJV: 64 (33.2%); RPS: 132 (67.3%)] consented to blood taking (**S1 Fig**).

### Anthropometric and hemoglobin assessments

The height (cm), weight (kg) and hemoglobin (Hb) concentration measurements were performed concurrently with the stool sample collection on site. For height, the respondent must be barefooted and was required to stand upright on a flat surface against the wall. A ruler was placed on top of the respondent’s head and a marker was used to mark the height. The height was measured from the mark on the floor with the aid of the measuring tape to the nearest 0.1 centimetres. Body weight was measured to the nearest 0.1 kilograms using a pre-calibrated locally manufactured weighing bathroom scale. Each respondent was required to stand on the scale with light clothing, without shoes, belts or other materials that could interfere with the actual reading. Height and weight measurements were taken by two trained field interviewers and recorded separately for accuracy. The average measurements for each participant were used for data analysis. Meanwhile, for Hb measurement, a cuvette was filled with capillary blood from a participant’s fingertips and analysed immediately with portable Diaspect (Sailauf, Germany) Hb measurement device.

### Nutritional and anemia profiles

For participants ≤19 years old, the anthropometric indices were analysed using WHO Anthro Plus software (version 3.2.2) to get the significant z-scores value for Height-for-Age (HAZ), Weight-for-Age (WAZ) (not calculated for children >10 years old) and BMI-for-age (BAZ). The values were assessed with reference to the National Centre for Health Statistics (NCHS) and WHO standards: overweight (> + 1SD BAZ z-score), obesity (> + 2SD BAZ z- score), wasting/thinness (< −2SD of BAZ z- score), underweight (<−2SD of WAZ z- score) and stunting (< −2SD of HAZ z-score). Therefore, children who had z-scores < -2SD of the NCHS/WHO reference data were considered as significantly undernourished [29]. Meanwhile, the body mass index (BMI) was used to reflect the nutritional status of the adult participants (> 19-year-old). It was calculated as body weight (kg) divided by the square of the height in meters (m^2^) regardless of age or sex. The cut-off points as defined by WHO are < 18.5 for underweight, 18.5–24.9 for normal, 25.0–29.9 for overweight and ≥ 30.0 for obesity.

We adapted the WHO threshold to define anemia as our standard reference [30]. Children age below than five years old and 12 years old were considered anemic if the recorded Hb level was below 11 g/dL and 11.5 g/dL, respectively. For participant age 12 and above and in non- pregnant women, Hb level <12 d/dL were considered to be anemic, while for men, it is below 13 g/dL. The Hb levels were then classified into levels of severity; mild, moderate and severe anemia as shown in **Table 1**[30].

**Table 1:** WHO cut-off values for anemia classification.

	Hemoglobin values in g/dL			
Age and gender	Non-Anemic	Mild	Moderate	Severe
Children < 5 years old	≥ 11.0	10.0–10.99	7.0–9.99	< 7
Children 5–11 years old	≥ 11.5	10.0–11.49	7.0–9.99	< 7
Children 12–14 years old	≥ 12.0	10.0–11.99	7.0–9.99	< 7
Pregnant women (>15)	≥ 11.0	10.0–10.99	7.0–9.99	< 7
Non-pregnant women (>15)	≥ 12.0	10.0–10.99	7.0–9.99	< 7
Men (> 15)	≥ 13.0	10.0–12.99	7.0–9.99	< 7

Adapted from World Health Organization, 2001.

### Data management and statistical analysis

Data were analysed using IBM SPSS version 21 (SPSS, Chicago, IL, USA). Demographic characteristics, i.e. gender, age groups, number of family members and household income were treated as categorical variables. Descriptive statistical analysis (frequency and percentages), measures of central tendency (means and medians) and dispersion (standard deviations, interquartile range) were computed to describe the characteristics of the studied population. The proportion with 95% confidence intervals (CI) was used to describe the prevalence. Continuous variables data were presented as either mean (95% CI) or mean (standard deviation) for normally distributed data and median (interquartile range, IQR) for non-normally distributed variables. Pearson Chi-square test (χ^2^) test was used to determine the association between categorical independent variables and the outcomes. For skewed distribution, Mann Whitney U-test was used to analyze mean differences of continuous variables (weight, height, and z-scores) according to STH infection status and other factors. A p-value of less than 0.05 (P ≤ 0.05) was considered significant.

Analysis on hemoglobin measurement, anemia status and their association with other variables was conducted amongst the 196 consented participants only. Since the distribution was normal, the independent t-test and one-way analysis of variance (ANOVA) were used to analyse the mean differences of Hb concentration between groups. Factors such as gender, age, number of family members and household income status which are probably associated with each undernutrition problems viz. underweight, stunting and wasting (among children and adolescent only) and anemia (all consented participants regardless of age group) were analysed by univariate and multivariate logistic regression (backward stepwise) and presented as the crude odds ratio (COR) and adjusted odds ratio (AOR) with 95% confidence interval (CI). For income status, although the cut-off threshold for poverty in rural area of Peninsular Malaysia is USD 206.5 [31] the monthly household income in this study was categorized into ~ ≤ USD 125 and > USD 125. This is because that more than half of the studied populations have a gross monthly income of less than ~ ≤ USD 125 (pre-determined as hardcore poverty). Apart from that, the association between soil-transmitted helminth infections and each undernutrition problems were also explored. Variables included in the analyses were those infected with specific STH infection (either *Trichuris trichiura*, *Ascaris lumbricoides* or hookworm) with the reference category of those who were found to be negative with the respective infection. The association between each STH species intensity (the burden of the STH infection per gram of feces) characterized by mild, moderate and severe infections [32] with undernutrition problems was also analyzed. However, variables with insufficient number of events (< 10) were not computed. Variables with P-value less than 0.25 [33] in univariate analysis were included for multivariate logistic regression. Model fitness was determined by the Goodness of fit statistical test.

## Results

### Anthropometric measurements and nutritional status among children and adolescents (≤ 19 y/o), N = 343)

#### Characteristics

We first analysed the anthropometric measurements and nutritional status (based on WAZ, HAZ and BAZ indices) of the Negrito children and adolescents. Of 416 overall participants, 343 (IJV: 117; RPS: 226) were children and adolescents aged between 2–19 years old with the mean age (95% CI) and median (IQR) of 8.5 (8.1, 8.9) and 9.0 (6.0, 11.0), respectively. Among them, 181 (52.8%) were males and 162 (47.2%) were females. Socio-demographic analysis revealed higher number of participants with the household income of >USD 125 in the IJV (36.8%) versus RPS (26.5%). Nevertheless, it is important to note that the cut-off threshold for poverty in rural area of Peninsular Malaysia is USD 206.5 [31]. By using this reference, 338 (81.3%) from the total participants has monthly household income of <USD 206.5 indicating that most of the Negritos were suffering from poverty. **Table 2**shows the characteristic of the children and adolescents included in this analysis.

**Table 2:** Socio-demographic characteristics of Negrito children and adolescents included in the anthropometric and nutritional assessments (N = 343).

Variables	Overall (N = 343)	IJV (N = 117)	RPS (N = 226)		
Demographic profiles	n (%)	n (%)	n (%)	χ^2^ (df)^a^	P value
**Gender**					
Male	181 (52.8)	67 (57.3)	114 (50.4)	1.4 (1)	0.23
Female	162 (47.2)	50 (42.7)	112 (49.6)		
**Exact age (Years)**					
Range	2.0–18.0	2.0–17.0	2.0–18.0	NA	NA
Median (IQR)	9.0 (6.0,11.0)	9.1 (8.4, 9.8)	8.3 (7.8, 8.7)		
Mean (95% CI)	8.5 (8.1, 8.9)	9.0 (6.0, 11.5)	8.0 (6.0, 11.0)		
**Age groups (Years)**					
2–5	71 (20.7)	20 (17.1)	51 (22.6)	1.8 (3)	0.61
6–8	97 (28.3)	34 (29.1)	63 (27.9)		
9–10	75 (21.9)	25 (21.4)	50 (22.1)		
11–19	100 (29.2)	38 (32.5)	62 (27.4)		
**Family members**					
≥ 7 members	233 (67.9)	77 (65.8)	156 (69.0)	0.4 (1)	0.55
< 7 members	110 (32.1)	40 (34.2)	70 (31.0)		
**Monthly income**					
> USD 125	103 (30.0)	43 (36.8)	60 (26.5)	3.8 (1)	0.05*
≤ USD 125	240 (70.0)	74 (63.2)	166 (73.5)		

^a^Pearson chi-square test (degree of freedom); P values were calculated to indicate the significant difference between the IJV and RPS; IQR = interquartile range; CI = confidence intervals; N = total number of participants; n = frequency; NA = not available;

*significant difference, **P ≤ 0.05.**

#### Anthropometric measurements (height and weight)

Overall analysis showed that the median body height was 97.0cm (2–5 y/o), 112.0cm (6–8 y/o), 124.0cm (9–10 y/o), and 141.0 cm (11–19 y/o). Meanwhile, the median body weight was 11.5kg in participants aged 2–5 years old (y/o), 16.0kg (6–8 y/o), 22.0kg (9–10 y/o), and 32.0kg in participants aged 11–19 years old. According to community categories, a comparable pattern of height (cm) and weight (cm) indices was generally observed between the age groups. However, the median height of children aged 9–10 y/o and 11–19 y/o and median weight of children aged 11–19 y/o were significantly higher in the IJV versus RPS **(S1 Table).**

#### Anthropometric measurements (HAZ, BAZ, WAZ)

The analyses demonstrated that the median z-score values for HAZ among children and adolescents aged 2–5 y/o and 6–8 y/o were within the normal range (> -2SD). However, lower median HAZ values which fall at the borderline of -2SD were noted in older groups of 9–10 y/o and 11–19 y/o Negritos. For both BAZ and WAZ, the lowest scores were perceived amongst the 6–8 y/o children and beyond the normal ranges of -2SD to < + 2SD.

Comparatively, no significant differences of median z scores were discovered between the IJV and RPS communities, except for IJV children aged 9–10 which had higher HAZ scores as compared to the same age group of children in RPS community (P = 0.01). Further analysis showed that the median z-scores of BAZ and WAZ were found to be crucial (< -2SD) among 6–8 y/o children in both communities, indicating the possibility of high incidence of acute undernutrition of wasting and underweight among them (**Table 3**).

**Table 3:** Comparison of HAZ, BAZ and WAZ profiles between the IJV and RPS Negritos (≤19 years old) according to age groups (N = 343).

Age range (Years)	Category		HAZ	BAZ	WAZ
		N	Median (IQR)	Median (IQR)	Median (IQR)
**2–5**	Overall	71	-1.0 (-2.6, 0.6)	-1.1 (-3.2, -0.1)	-2.0 (-2.9, -1.2)
	IJV	20	-0.7 (-2.7, 0.8)	-1.7 (-3.1, -0.2)	-1.6 (-2.7, -1.0)
	RPS	51	-1.3 (-2.6,0.5)	-1.1 (-3.7, -0.1)	-2.1 (-3.1, -1.2)
	**P value**		0.64	0.86	0.48
**6–8**	Overall	97	-1.7 (-2.5, -0.7)	-2.3 (-3.6, -1.2)	-2.7 (-3.5, -1.9)
	IJV	34	-1.3 (-2.5, -0.7)	-2.3 (-3.5, -1.0)	-2.3 (-3.5, -1.6)
	RPS	63	-1.9 (-2.7, -0.6)	-2.3 (-3.6, -1.3)	-2.9 (-3.6, -2.2)
	**P value**		0.44	0.75	0.21
**9–10**	Overall	75	-2.0 (-2.8, -1.4)	-1.2 (-2.3, 0.3)	-1.9 (-3.1, -1.9)
	IJV	25	-1.5 (-2.0, -1.3)	-0.8 (-2.3, 0.1)	-1.8 (-2.5, -0.7)
	RPS	50	-2.2 (-3.0, -1.4)	-1.2 (-2.3, 0.5)	-2.1 (-3.4, -0.6)
	**P value**		0.01*	0.87	0.33
**11–19**	Overall	100	-2.0 (-2.6, -1.0)	-1.3 (-1.9, -0.5)	NA
	IJV	38	-2.1 (-2.6, -1.0)	-1.1 (-1.7, -0.5)	
	RPS	62	-1.9 (-2.7, -1.5)	-1.4 (-2.1, -0.5)	
	**P value**		0.50	0.40	

N = Number of examined participants; P values were calculated based on Mann-Whitney U test to indicate the significant difference between the IJV and RPS communities; IQR = interquartile range); HAZ = height-to-age Z score; BAZ = BMI-to-age Z score; WAZ = weight-to-age Z score; NA = not available, since WAZ reference values are only suitable up to 10 years old;

*significant different, **P ≤ 0.05.**

#### Prevalence of undernutrition (stunting, wasting and underweight)

The prevalence of undernutrition was further calculated. Of the total of 343, 248 (72.3%; 95% CI: 67.6, 77.0) children and adolescent Negritos were found to be afflicted with some form of undernutrition problems (i.e. stunting, wasting and underweight) (**Table 4**). The overall prevalence of stunting, wasting, and underweight were 45.8%, 42.3% and 59.1%, respectively.

**Table 4:** Prevalence of stunting, wasting and underweight amongst children and adolescent Negritos.

Category	Overall (N = 343)		IJV (N = 117)		RPS (N = 226)		
	n (%)	95% CI	n (%)	95%CI	n (%)	95% CI	P value^a^
No. of participants with any form of undernutrition^¥^	248 (72.3)	67.6, 77.0	80 (68.4)	60.0, 76.8	168 (74.3)	68.6, 80.0	0.24
**HAZ**^**b**^	N = 332		N = 112		N = 220		0.03*
Normal (-2SD to < +2SD)	180 (54.2)	48.8, 59.6	66 (58.9)	49.9, 68.3	114 (51.8)	44.9, 58.3	
Stunting (-3SD to < -2SD)	104 (31.3)	26.4, 36.5	38 (33.9)	24.8, 42.4	66 (30.0)	24.1, 36.3	
Severe stunting (< -3SD)	48 (14.5)	10.7, 18.3	8 (7.1)	2.4, 12.2	40 (18.2)	13.0, 23.3	
**BAZ**^**b**^	N = 319		N = 111		N = 208		0.26
Normal (-2SD to <+2SD)	184 (57.7)	52.3, 63.1	70 (63.1)	54.1, 72.1	114 (54.8)	48.0, 61.6	
Wasting (-3SD to < -2SD)	70 (21.9)	17.4, 26.4	19 (17.1)	10.1, 24.1	51 (24.5)	18.7, 30.3	
Severe wasting (< -3SD)	65 (20.4)	16.0, 24.8	22 (19.8)	12.4, 27.2	43 (20.7)	15.2, 26.2	
**WAZ**^**b**^^≠^	N = 237		N = 77		N = 160		0.03*
Normal (-2SD to <+2SD)	97 (40.9)	34.6, 47.2	39 (50.6)	39.4, 61.8	58 (36.3)	28.9, 44.1	
Underweight (-3SD to <-2SD)	69 (29.1)	23.3, 34.9	23 (29.9)	19.7, 40.1	46 (28.8)	21.8, 35.8	
Severe underweight (< -3SD)	71 (30.0)	24.2, 35.8	15 (19.5)	10.7, 28.4	56 (35.0)	27.6, 42.4	
**Stunting + Wasting**	47 (18.9)	14.0, 23.8	11 (13.8)	6.2,21.4	36 (21.4)	15.2, 27.6	0.08

N = Number of examined participants; n = frequency;

^¥^Stunting, wasting or underweight;

^a^ P values were calculated based on Pearson chi-square (χ^2^) test to indicate the significant difference between the IJV and RPS communities;

^b^ Children with > +2SD for every indicator were excluded from the analyses resulted in different number of N as indicated in the table; ≠ WAZ reference values are only suitable up to 10 years old; CI = confidence intervals;

*significant different, **P ≤ 0.05.**

By community categories, the prevalence of any types of undernutrition was found to be slightly higher in the RPS (74.3%) as compared to IJV (68.4%), however the finding was not significant (P = 0.24). Stratified by each problem, the prevalence of stunting (HAZ), wasting (BAZ) and underweight (WAZ) were 48.2%, 45.2% and 63.8% in the RPS, while 41.0%, 36.9% and 49.4% in the IJV, (P = 0.10, 0.15, and 0.03), respectively.

Interestingly, stunting (chronic form of undernutrition) was more critical in the RPS as the prevalence of stunting and severe stunting (HAZ scores < -2SD and < -3SD) were 30.2% and 18.1% in the RPS versus 33.6% and 7.3% in the IJV (33.6%; 7.3%), respectively (P = 0.03). Meanwhile, no significant difference was seen in the prevalence of wasting (acute form of undernutrition) and severe wasting (BAZ scores < - 2SD and < -3 SD) in the RPS (24.5%; 20.7%) compared to IJV (17.1%; 19.8%) (P = 0.26). Nevertheless, 36 (21.4%) RPS and 11 (13.8%) IJV children and adolescents were found to be suffering from both stunting and wasting (P = 0.08). The issue of underweight was also found to be more significant amongst the RPS, characterized by the prevalence of severe underweight (WAZ score < -3SD) of 35.0% as compared to the 19.5% in the IJV (P = 0.03).

### Anthropometric measurements and nutritional status among adult Negritos (> 19 y/o), N = 73)

Of 73 (IJV:32; RPS:41) paired data, 27 (37.0%) were males and 46 (63.0%) were females. Their age ranged from 20.0–64.0 with mean age and median of 30.4 (95% CI: 27.8, 33.0) and 29.0 (IQR: 21.0, 37.0), respectively (**S2 Table**). The overall median body weight (kg), body height (cm) and body mass index, BMI (kg/m^2^) were 49.0 (IQR: 46.0, 54.5), 151.0 (IQR: 147.0, 156.0) and 21.4 (IQR: 19.6, 25.1), respectively. Further nutritional assessment revealed that majority of the adults (> 65.0%) had normal BMI values.

Comparatively, no significant difference was discovered for all mean indices of anthropometric measurement and nutritional status amongst the IJV and RPS adults. The prevalence of underweight was relatively low (< 7.5%) in both groups. However, overweight and obese were found to be surprisingly prominent (19/73; 26.0%) and afflicted mostly the females (>80%) in both communities.

### Hemoglobin (Hb) concentration and anemia status (N = 196)

#### Hemoglobin profiles

Of the 416 total participants, only 196 (IJV: 64; RPS: 132) consented for Hb test. From this number, 107 (54.6%) were females and 89 (45.4%) were males. Majority (108, 55.0%) of the participants were school-aged children of 7–12 years old, 40 (20.4%) aged 2–6 years old and 48 (24.5%) aged ≥ 13 years old. Altogether, the mean Hb level was 10.8g/dL (95% CI: 10.5, 11.0).

Comparatively, the mean Hb concentration level was comparable in both communities [IJV: 10.9g/dL (95% CI: 10.5, 11.2); RPS: 10.7g/dL (95% CI: 10.4, 11.0)], (P = 0.43). Stratification by gender showed comparable findings between the mean Hb of the females and males, except in IJV where the females had significantly higher value of mean Hb [females: 11.3g/dL (95% CI: 10.8, 11.9); males: 10.5g/dL (95% CI: 10.1, 10.9)] (P = 0.02). Nevertheless, the variance in mean Hb values was significant between the age groups and increased with advancing age (the lowest among those aged 2–6 and the highest in those ≥ 13 years old) in both communities (**S3 Table**).

#### Anemia status

Overall prevalence of 68.4% (134/196; 95% CI: 61.9, 74.9) for anemia was discovered among the consented Negritos. By community categories, the prevalence was comparable between the RPS community [69.7% (95% CI: 44.9, 77.2)] and in the IJV [65.6% (95% CI: 57.8, 73.4)] (P = 0.57). By gender, no significant differences were observed between the male and female participants in both communities. However, the prevalence of anemia was found to be the highest among young children aged 2–6 years (80.0%: 95% CI: 67.6, 92.4) with decreased trend as age group advances in both communities (**Table 5**). It is important to highlight that approximately half of the anemic participants had moderate to severe anemia status (40.5% in the IJV and 47.8% in the RPS). Four children had severe anemia and all of them were from the RPS community.

**Table 5:** Prevalence of anemia amongst the IJV and RPS Negritos.

Variables	Overall (N = 196)		IJV (N = 64)		RPS (N = 132)		P value^b^
**Anemia status**^**a**^	**n (%)**	**95% CI**	**n (%)**	**95% CI**	**n (%)**	**95% CI**	
Non-anemia	62 (31.6)	25.1, 38.1	22 (34.4)	22.8, 46.0	40 (30.3)	22.5, 38.1	0.57
Overall anemia	134 (68.4)	61.9, 74.9	42 (65.6)	44.9, 77.2	92 (69.7)	57.8, 73.4	
**Gender**							
Male	64 (71.9)	62.6, 80.8	24 (72.7)	60.3, 87.9	40 (71.4)	59.6, 83.2	0.89
Female	70 (65.4)	56.4, 74.4	18 (58.1)	40.7, 75.5	52 (68.4)	58.0, 78.9	0.31
P value^a^	0.33		0.22		0.71		
**Age groups**							
2–6	32 (80.0)	67.6, 92.4	14 (82.4)	64.3, 100	18 (78.3)	61.4, 95.2	0.54
7–12	76 (70.4)	61.8, 79.0	16 (57.1)	38.8, 75.4	60 (75.0)	66.0,84.1	0.08
≥13	26 (54.2)	40.1, 68.3	12 (63.2)	41.5, 84.9	14 (48.3)	30.1, 66.5	0.31
P value^a^	0.03*		0.22		0.02*		
**Severity of anemia**							0.34
Mild	73 (54.5)	46.7, 67.9	25 (59.5)	46.1, 74.3	48 (52.5)	42.3, 62.7	
Moderate	57 (42.5)	34.1, 50.9	17 (40.5)	25.7, 55.4	40 (43.5)	33.4, 53.6	
Severe	4 (3.0)	0.1, 5.9	0		4 (4.3)	0.2,8.5	

^a^P value was calculated based on chi-square test between variable;

^b^P value was calculated based on chi-square test between category community; n = frequency; CI = confidence interval;

*significant different, **P ≤ 0.05.**

### Associations between malnutrition with STH infections and other variables

#### Soil-transmitted helminth (STH) infections amongst the children and adolescents

The comprehensive status of soil-transmitted helminth (STH) infections amongst the overall Negritos have been published [26]. However, only data related to children and adolescents (≤ 19 y/o) was included. In brief, a total of 292 (85.1%) were found to be infected with at least one of the STH parasites. According to STH species, 244 (71.1%), 159 (46.4%) and 68 (19.8%) children and adolescent were positive with *T*. *trichiura*, *A*. *lumbricoides* and hookworm, respectively. A total of 143 participants were inflicted with single STH infection (mono-parasitism, regardless of STH species) and 149 had STH poly-parasitism characterized mainly by double infection of *T*. *trichiura* and *A*. *lumbricoides*.

#### Potential factors associated with undernutrition (underweight, stunting, wasting)

The association between undernutrition and STH infections with other variables were computed amongst all children and adolescents aged ≤19 y/o (N = 343), except for WAZ which only concerned children aged ≤ 10 y/o (N = 243). Univariate analysis revealed that children aged ≤ 10 years old who lived in the RPS had 1.8 odds in suffering from underweight problems compared to those in the IJV (P = 0.03). Meanwhile, 1.3 and 1.4 odds of being stunted and wasted were observed in the RPS versus the IJV but with no significant difference (P values = 0.22 and 0.16, respectively).

We then specifically analysed (univariate and multivariate logistic regression) the eleven possible factors which might be associated with each case of underweight (WAZ), stunted (HAZ), and wasted (BAZ) in both the IJV and RPS communities.

*A) Potential factors associated with underweight*. In the IJV community, those infected with STH poly-parasitism and children aged ≤ 6 had 3.7 (95% CI: 1.3, 10.2; P = 0.02) and 3.2 (95% CI: 1.1, 9.3; P = 0.03) odds for being underweight [Goodness of fit: X^2^ = 0.1 (df = 2); P = 0.95] (**Table 6**). Meanwhile, in the RPS community (**Table 7**), those who had large family members of > 7, being infected with the *T*. *trichiura* infection and being infected with STH polyparasitism increased the risk of underweight by 2.0, 3.1 and 2.3 odds, respectively. However, further multivariate findings retained only *T*. *trichiura* infection (P = 0.04) and STH poly-parasitism (P = 0.05) as the significant predictors for being underweight.

**Table 6:** Potential risk factors associated with underweight (WAZ < -2SD) in the IJV community (univariate and multivariate logistic regression), N = 77.

Variables	N	Underweight n (%)	Univariate COR (95% CI)	P value	Multivariate AOR (95% CI)	P value
Male	42	20 (47.6)	1.2 (0.5, 2.9)	0.74	**	**
Female	35	18 (51.4)	1			
Age ≤ 6#	29	18 (62.1)	2.3 (0.9, 5.9)	0.08*	3.2 (1.1, 9.3)	0.03*
Age > 6	48	20 (41.7)	1			
Family member ≥7	49	25 (51.0)	1.2 (0.6, 2.0)	0.70	**	**
Family member <7	28	13 (46.4)	1			
Income < RM500	27	26 (52.0)	1.4 (0.5, 3.5)	0.53	**	**
Income ≥ RM500	50	12 (44.4)	1			
Infected (TT)	59	32 (54.2)	nc	nc	nc	nc
Negative	18	6 (33.3)				
Moderate-severe TT*#*	39	22 (56.4)	1.8 (0.7, 4.4)	0.21	1.0 (0.3, 2.9)	0.99
Negative-mild	38	16 (42.1)	1			
Infected (AL)*#*	33	20 (60.6)	2.2 (0.9, 5.6)	0.09	1.0 (0.26, 4.1)	0.96
Negative	44	18 (40.9)	1			
Moderate-severe AL	21	11 (52.4)	1.2 (0.4, 3.2)	0.75	**	**
Negative-mild	56	27 (48.2)	1			
Infected (Hkw)	18	11 (61.1)	1.9 (0.6, 5.5)	0.25	1.0 (0.2, 4.4)	0.97
Negative	59	27 (45.8)	1			
Moderate-severe Hkw	7	4 (57.1)	nc	nc	nc	nc
Negative-mild	70	34 (48.6)				
STH Poly-parasitism#	32	22 (68.8)	3.7 (1.4, 9.8)	0.01*	3.7 (1.3, 10.2)	0.02*
STH Mono-parasitism	40	15 (37.5)	1			

N = number of examined participants; (1) reference variable; COR (95% CI): Crude odd ratio (95% confidence interval); AOR: Adjusted odd ratio; TT = *T*. *trichiura*; AL = *A*. *lumbricoides*; Hkw = Hookworm;

#Variable included in the logistic multivariate regression analysis because the P value of COR was < 0.25.

** No value is available because the variables were not included in the multivariate analysis; nc: Not computed due to insufficient events per variables of < 10;

*significant different, **P ≤ 0.05.**

**Table 7:** Potential risk factors associated with underweight (WAZ < -2SD) in the RPS community (univariate and multivariate logistic regression), N = 160.

Variables	N	Underweight n (%)	Univariate COR (95% CI)	P value	Multivariate AOR (95% CI)	P value
Male #	80	56 (70.0)	1.7 (0.9, 3.3)	0.10	1.9 (0.9, 4.2)	0.09
Female	80	46 (57.5)	1			
Age ≤6	69	42 (60.9)	1.2 (0.7, 2.4)	0.51	**	**
Age >6	91	60 (65.9)	1			
Family member ≥7#	114	78 (68.4)	2.0 (1.0, 4.0)	0.05*	1.9 (0.8, 4.4)	0.13
Family member <7	46	24 (52.2)	1			
Income <RM500#	123	82 (66.7)	1.7 (0.8, 3.6)	0.16	1.5 (0.6, 4.1)	0.41
Income ≥RM500	37	20 (54.1)	1			
Infected (TT)#	113	81 (71.7)	3.1 (1.5, 6.3)	0.002*	2.8 (1.0, 7.6)	0.04*
Negative	47	47 (44.7)	1			
Moderate-severe TT*#*	80	59 (73.8)	2.4 (1.2, 4.7)	0.01*	1.4 (0.6, 3.6)	0.46
Negative-mild	80	43 (53.8)	1			
Infected (AL)#	75	53 (70.7)	1.8 (0.9, 3.4)	0.09	1.3 (0.4, 5.6)	0.71
Negative	85	49 (57.6)	1			
Moderate-severe AL	57	38 (66.7)	1.2 (0.6, 2.4)	0.57	**	**
Negative-mild	103	64 (62.1)	1			
Infected (Hkw)	23	16 (69.6)	1.4 (0.5, 3.5)	0.53	**	**
Negative	137	86 (62.8)	1			
Moderate-severe Hkw	5	2 (40.0)	nc	nc	nc	nc
Negative-mild	155	100 (64.5)				
STH Poly-parasitism#	69	53 (76.8)	2.3 (1.1, 4.8)	0.03*	2.2 (1.0, 4.8)	0.05*
STH Mono-parasitism	64	38 (59.4)	1			

N = number of examined participants; (1) reference variable; COR (95% CI): Crude odd ratio (95% confidence interval); AOR: Adjusted odd ratio; TT = *T*. *trichiura*; AL = *A*. *lumbricoides*; Hkw = Hookworm;

#Variable included in the logistic multivariate regression analysis because the P value of COR was < 0.25.

** No value is available because the variables were not included in the multivariate analysis; nc: Not computed due to insufficient events per variables of < 10;

*significant different, **P ≤ 0.05.**

*B) Potential factors associated with stunting*. In the IJV community, univariate analysis showed that stunting was significantly higher among older children aged > 10 y/o versus those ≤ 10 years old [COR: 2.3, 95% CI: 1.0, 5.1 (P = 0.04)]. No other significant association with STH infections was further observed in IJV (**S4 Table**). Meanwhile, in RPS, univariate and multivariate analyses indicated that being infected with STH poly-parasitism was the significant risk factor for stunting [(AOR: 1.9 (95% CI: 1.0, 3.4) (Goodness of fit: X^2^ = 3.3 (df = 5); P = 0.66) (**S5 Table**).
*C) Potential factors associated with wasting*. In the IJV community, both the univariate and final multivariate analyses indicated that only the age group of ≤ 10 years old (P < 0.001) and low monthly household income (P = 0.01) were the only important variables for determining wasting [Goodness of fit: X^2^ = 0.01 (df = 2); P = 0.99] (**S6 Table**), whereas in the RPS community (**S7 Table**), being a male and children aged ≤ 10 years old were found as significant predictors for wasting (multivariate analysis: AOR 2.3, P = 0.003; AOR 2.6, P = 0.002, accordingly) [Goodness of fit: X^2^ = 0.15 (df = 2); P = 0.93]. No direct association was found between wasting with any STH infections in both IJV and RPS communities.

#### Potential factors associated with anemia

The association between anemia and thirteen selected factors were first analysed independently (univariate logistic regression). In the IJV (**Table 8**), being infected with *A*. *lumbricoides *[COR: 3.4 (95% CI: 1.0, 11.7), P = 0.04], being infected with moderate-to-severe *T*. *trichiura* [COR: 4.1 (95% CI: 1.3, 13.2), P = 0.01] and being positive with STH poly-parasitism [COR: 8.1 (95% CI: 1.6, 39.9), P = 0.004] were the significant risk factors for anemia. However, from six variables entered in the multivariate analysis, only *A*. *lumbricoides* [(AOR: 5.7 (95% CI: 1.6, 20.8), P = 0.01] and moderate-to-severe *T*. *trichuira* infections [AOR: 4.7 (95% CI: 1.2, 18.6), P = 0.03] retained as significant predictors [Goodness of fit: X^2^ = 3.4 (df = 6); P = 0.75].

**Table 8:** Potential factors associated with anemia amongst the IJV Negritos (logistic univariate and multivariate regression), N = 64.

Variables	N	Anemic Negritos, n (%)	COR (95% CI)	P value	AOR (95% CI)	P value
Male ^#^	33	24 (72.7)	1.9 (0.7, 5.5)	0.22	2.8 (0.8, 10.4)	0.12
Female	31	18 (58.1)	1			
Age <9	32	22 (68.8)	1.3 (0.5, 3.7)	0.60	**	**
Age ≥9	32	20 62.5)	1			
Family member ≥7	47	30 (63.8)	0.7 (0.2, 2.4)	0.62	**	**
Family member <7	17	12 (70.6)	1			
Income <RM500	33	20 (60.6)	0.6 (0.2, 1.8)	0.38	**	**
Income ≥RM500	31	22 (71.0)	1			
Stunted	21	14 (66.7)	0.9 (0.3, 2.9)	0.86	**	**
Normal	29	20 (69.0)	1			
Wasted	17	11 (64.7)	0.9 (0.3, 3.0)	0.83	**	**
Normal	34	23 (67.6)	1			
Underweight	18	13 (72.2)	1.5 (0.4, 6.1)	0.56	**	**
Normal	19	12 (63.2)	1			
Infected (TT)	45	29 (64.4)	0.8 (0.3, 2.6)	0.76	**	**
Negative	19	13 (68.4)	1			
Moderate-severe TT^*#*^	22	18 (81.8)	3.4 (1.0, 11.7)	0.04*	4.7 (1.2, 18.6)	0.03*
Negative-mild	42	24 (57.1)	1			
Infected (AL)^*#*^	28	23 (82.1)	4.1 (1.3, 13.2)	0.01*	5.7 (1.6, 20.8)	0.01*
Negative	36	19 (52.8)				
Moderate-severe AL^*#*^	17	14 (82.4)	3.2 (0.8, 12.5)	0.09	1.9 (0.2, 16.9)	0.56
Negative-mild	47	28 (59.6)				
Infected (Hkw)	8	7 (87.5)	nc	Nc	nc	nc
Negative	56	35 (62.5)				
STH Poly-parasitism^#^	20	18 (90.0)	8.1 (1.6, 39.9)	0.004*	1.8 (0.2, 15.5)	0.62
STH Mono-parasitism	38	20 (52.6)	1			

N = number of examined participants; (1) Reference variable; COR (95% CI): Crude odd ratio (95% confidence interval); AOR: Adjusted odd ratio;

#Variable included in the logistic multivariate regression analysis because the P value of COR was <0.25.

** No value is available because the variables were not included in the multivariate analysis; nc: Not computed due to insufficient events per variables of ≤10;

*Significant finding of **P≤0.05.**

In contrast, there was no significant relationship between any STH infections and anemia among RPS Negritos (**Table 9**). Only stunted individual was found as a potential risk factor and retained as a significant predictor for anemia in multivariate analysis in this community [AOR: 7.1 (95% CI: 2.0, 24.9); P = 0.002). [Goodness of fit: X^2^ = 4.8 (df = 5); P = 0.44].

**Table 9:** Potential factors associated with anemia amongst the RPS Negritos (logistic univariate and multivariate regression), N = 132.

Variables	N	N (%)	COR (95% CI)	P value	AOR (95% CI)	P value
Male	56	40 (71.4)	1.2 (0.5, 2.5)	0.71	**	**
Female	76	52 (68.4)	1			
Age < 9^#^	62	48 (77.4)	2.0 (0.9, 4.4)	0.07	3.3 (0.8, 13.3)	0.09
Age ≥ 9	70	44 (62.9)	1			
Family member ≥7^#^	86	57 (66.3)	0.6 (0.3, 1.4)	0.24	0.5 (0.1, 1.5)	0.20
Family member <7	46	35 (76.1)	1			
Income < RM500^#^	83	54 (65.1)	0.5 (0.2, 1.2)	0.13	0.7 (0.2, 2.4)	0.57
Income ≥ RM500	49	38 (77.6)	1			
Stunted^#^	62	53 (85.5)	4.4 (1.8, 11.3)	0.001*	7.1 (2.0, 24.9)	0.002*
Normal	44	25 (56.8)	1			
Wasted	41	28 (68.3)	0.7 (0.3, 1.7)	0.43	**	**
Normal	61	46 (75.4)	1			
Underweight^#^	28	18 (64.3)	0.5 (0.2, 1.5)	0.21	0.7 (0.2, 2.9)	0.63
Normal	49	38 (77.6)	1			
Infected (TT)	103	71 (68.9)	0.8 (0.3, 2.1)	0.72	**	**
Negative	29	21 (72.4)	1			
Moderate-severe TT	67	47 (70.1)	1.0 (0.5, 2.2)	0.91	**	**
Negative-mild	65	45 (69.2)	1			
Infected (AL)	64	46 (71.9)	1.2 (0.6, 2.6)	0.60	**	**
Negative	68	46 (67.6)	1			
Moderate-severe AL	51	37 (72.5)	1.2 (0.6, 2.7)	0.57	**	**
Negative-mild	81	55 (67.9)	1			
Infected (Hkw)	25	18 (72.0)	1.1 (0.4, 3.1)	0.78	**	**
Negative	107	74 (69.2)	1			
STH Poly-parasitism	69	49 (71.0)	1.2 (0.5, 2.7)	0.83	**	**
STH Mono-parasitism	40	27 (67.5)	1			

N = number of examined participants; (1) Reference variable; COR (95% CI): Crude odd ratio (95% confidence interval); AOR: Adjusted odd ratio;

#Variable included in the logistic multivariate regression analysis because the P value of COR was <0.25.

** No value is available because the variables were not included in the multivariate analysis; nc: Not computed due to insufficient events per variables of ≤10;

*Significant finding of **P≤0.05.**

## Discussion

In this study, the current nutritional and anemia status, and their associations with STH infections and other selected variables amongst the present-day hunter gatherer of Negritos living in the inland forest (IJV), and in resettlements closer to the town peripheries (RPS) were determined. Overall, the undernutrition problems based on HAZ, BAZ and WAZ were found to be highly crucial amongst children and adolescent Negritos stipulated by 45.8% prevalence of stunting (chronic), 42.3% of wasting (acute) and 59.1% of underweight. These figures were ~ 3–4 times higher than the undernutrition problem amongst general Malaysian averages (13.7%– 20.7%) [8]. In addition, the prevalence rates in these Negrito community were also relatively greater than prevalence of underweight (29.2%– 56.5.3%) and wasting (12.5%-30.0%) reported in other indigenous tribes, i.e., the Senoi and Proto-Malay. However, the prevalence for stunting among the Negrito was within similar ranges reported in other indigenous tribes [4–7].

Contrary to the findings observed in the children and adolescents, low prevalence of underweight (6.8%) with majority having normal BMI value were discovered amongst the adult Negritos. This scenario is in agreement with previous report [34] whereby the adults especially males are less susceptible to undernourishment and could tolerate a higher proportion of mass body reduction as compared to the children [35]. However, our study also showed the increased prevalence of overweight and obese (26.0%) particularly amongst the adult females. Although our present scope did not specifically measure the double-burden of households (overweight mother/underweight children), this present finding indirectly suggested the possibility of the double-burden of malnutrition phenomenon among the Negritos and it is an emerging problem which has been highlighted earlier in other tribes [36]. The prevalence of overweight and obesity among adult females of the Senoi and Proto Malay living in semi-urbanised areas [37] was reported to be two-fold higher than the findings from our study. Low food variety score was argued as a potential factor for this problem.

This present study also revealed a high prevalence (68.4%) of anemia, with 80% and 70.4% of Negrito children aged 2–6 y/o and aged 7–12 y/o were found to be anemic, respectively. This figure was relatively higher than the ranged of prevalence reported in other OA tribes (26.2%-48.5%) [4,9,38]. According to age, the number of anemic participants gradually decreased in older and adult participants and this is consistent with the findings of earlier local studies [4,38]. Meanwhile, gender stratification in this study showed no difference between the incidence of anaemia between the females and males although it is well acknowledged that females tend to be more anemic as a result of physiological differences [39].

In the comparative analysis, we found that the prevalence of underweight (WAZ) were significantly higher in the children and adolescents in RPS (63.8%) versus their IJV counterparts (and 49.4%). Whilst, the prevalence of anemia was found to be comparable in the RPS (69.7%) and IJV (65.6%). Although the significant difference was only observed in underweight problem (concerned only among children ≤ 10 y/o), more than 20% of the RPS children suffered from the co-occurrence of wasting and stunting. These findings showed the malnutrition problem among the RPS community was grave as compared to the IJV despite living nearer to the mainstream population and expose to rapid demarginalization.

Further findings showed that the persistence of STH-infections was likely associated or has aggravated the undernutrition and anemia problems. In the IJV, apart from being children aged ≤ 6, STH poly-parasitism was also found to be the determinant for significant underweight (WAZ) problems. No significant association was observed between being underweight with specific parasitic infection (*T*. *trichiura*, *A*. *lumbricoides* or hookworm). These findings are possibly correlated with the postulation on the role of parasitic infections as a natural controller of the population size among the hunter-gatherer indigenous community who lived in a diverse tropical rain forest ecosystem [40]. The nature of host-parasite relationship which commonly manifested in conditions such as severe helminth infections and poor health condition of the human hosts ultimately supports the above concept [41] and our current findings. Hence, even if not directly associated, we could postulate the existence of some synergistic effects or “the survival of the fittest” theory among the IJV individuals suffering from the highest numbers of pathogens (poly-infections) and underweight problems, which could lead to mortality, especially among children.

Conversely, in the RPS, underweighted problems were found to be significantly associated with *T*. *trichiura* infection. This result is expected as there is a higher prevalence and intensity of *T*. *trichiura* in the RPS community versus the IJV as described in a previous study [26]. Despite mass anti-helminthic treatment (AHT) being administered in the RPS, *T*. *trichiura* has been acknowledged as the most predominant parasite with a fast re-infection rate (1–3 months) in most of the resettled RPS community in Malaysia.

With regard to wasting and stunting, both indicators share common risk factors (e.g. nutritional deficiencies, diarrhoea, and infection) and effects (e.g. delayed cognitive and psychomotor development), which, if persistent, would synergistically lead to higher mortality risk [42]. In this study, no direct association was found between wasting and STH infections in both IJV and RPS Negritos except higher in participants with low household income status and in male children aged ≤ 10.

Unlike wasting (acute), stunting is commonly seen as a chronic manifestation resulting from prolonged undernourishment and repetitive infections. Therefore, stunting was expected to be prominent amongst the older children compared to younger age group as reported elsewhere [43,44]. This likely association was significantly observed in the IJV as those aged 10–19 y/o had 2.3 odds to be stunted versus those children aged ≤10. Conversely, such relationship was not pronounced in their RPS counterpart. The only significant predictor for stunting in the RPS community was STH polyparasitism, partly supporting the role of multiple infections in chronic undernutrition problem. As a whole, the present observations need to be translated carefully since the Negritos are generally small physically, and short stature, with most measuring five feet or less [45]. In the evolutionary biology of hunter-gatherers, the unique lifestyle of the OA due to long adaptations in isolated forest environment has probably shaped their physical and biological outcomes [41]. Small stature has also been noticed in other hunter-gatherer populations of Africa (Hadza, Twa Pygmies, and San) and among Andaman Islanders [41]. A similar result was obtained in this study, as the average height of the adult Negritos was less than ~152cm. Therefore, apart from nutritional deficiencies and STH infections, their stunting or small size could be a deliberate genetic trait. This characteristic allows them to be more mobile and agile as former nomadic hunter-gatherers in the rainforest, and hence requiring lesser energy for survival during shortage of food resources and more efficient in hunting and foraging activities.

We also highlighted that the moderate and severe intensity of *T*. *trichiura* infection contributed to ~4 to 5 times risk of anemia as compared to those with negative-light infections in the IJV. In contrast, being stunted was indicated as a significant risk factor for anemia in the RPS. The inter-relation between these two problems could be a result of prolong persistence of STH infections in the RPS, which leads to further stunting and subsequently low hemoglobin levels among them.

Taking these results together, evidence from this present study has highlighted some failures of demarginalization, due to environmental-cultural changes after ~ 40 years of redevelopment and resettlement while embracing modernity. As former hunter-gatherers, the changes that they have adopted from a traditional to a new modern lifestyle is considered drastic. The dietary habits of OA have been drastically changed from subsistence cropping, forest plant and wildlife resources to commercial and processed food dependency (e.g. sweetened milk creamer, sugar and wheat flour), following resettlement [18]. This nutritional transition could lead to low dietary diversity score and consequently nutritional deficiencies especially given their low purchasing power. The predicament of double-burden of malnutrition may also increase if attention on nutrition shifts from traditional diets to contemporary foods high in sugars and fat are being neglected [46]. This issue deserves attention, because it might be interrelated to high predisposition to cardiovascular diseases and insulin resistance in the indigenous people as reported recently in Malaysia [47].

Relocation to unfavourable location as experienced particularly amongst the RPS Negritos, might very well aggravate the situation whenever access to traditional forest land which has had a vital role in their diets have been abandoned or reduced. The inability to cope with the new environmental areas and the inadequate production of cash crop agriculture may cause the OA to fall into deeper level of poverty, resulting in shortage of adequate food supply. This indirectly reflects the higher status of undernutrition and anemia among children and adolescents in this community. Unlike RPS, the IJV community which are currently undergoing the *in-situ* development might have some advantages. In the event of food supply shortage, the IJV people still have broader access to the forested area to forage for adequate food resources and medicine. This speculation is supported by previous findings which indicated high dietary diversity score (DDS) among the OA involved in collecting forest products from their surrounding areas [20].

Nevertheless, although the *in-situ* development may benefit the IJV Negrito, their undernutrition problem is considered crucial as the RPS; and their fate would become similar to the RPS if effective measures are not taken to arrest the current situation. The most probable reasons are revolving around factors such as progressive deforestation and extensive logging which could contaminate water reservoirs and cause forest ecosystem imbalance which is fundamental for their subsistence. Definite modernisation and losing power over their traditional land might consequently force the IJV community to change their lifestyle drastically.

Meanwhile, the STH-associated malnutrition poses a significant threat to the survival, physical growth, and cognitive development, especially among the younger OA generation. This problem has been highlighted in these recent years and more likely to occur among OA children with high burden of STH infection. In the past, nomadic lifestyles were known to hinder communities from being afflicted with significant amount of infections [19,48], hence reducing the risk of STH-associated malnutrition.

Adoption of similar habits such as open defecation and poor hygiene behaviours in crowded resettlement or modified inhabitant areas have been suggested to partly contribute to the increased loads of intestinal parasites [26]. Sedentary lifestyles have further exacerbated the situation through frequent contact with fecal contamination in soil. It was acknowledged that many STH infections affect the appetite and consequently reduce the food intakes and may persist even during the recovery phase [49–51]. STH infections have also been reported to be connected to severe protein-energy malnutrition especially among children [10]. These findings need to be addressed urgently since the risk of morbidity and mortality multiplies with the existence of both problems among younger individuals [40]. If left untreated, they would synergistically cause the overall health of the OA community to deteriorate and this will impact their immune system’s ability to fight against more life-threatening introduced pathogens following demarginalization [16,25].

Some limitations in the current study need to be considered while interpreting the present findings. Firstly, the data and results were cross-sectional or based on one time point only. A longitudinal study will be beneficial in the future for causal effects and to understand the association between infections and malnutrition over a period of time. Secondly, although the Negrito villages was randomly selected based on the list given by the authorities, sampling at individual level was based on convenient, snowball method and this may provide some biased outcomes, as the sampling is not random. Nevertheless, the sampling approach was the most appropriate and feasible considering the logistics challenges to reach the Negritos especially in the IJV community. Thirdly, the number of adult participants was low due to a high refusal rate to participate in the study. Fourth, the measurements for the undernutrition status were based on the reference standard by the WHO which does not include indigenous or pygmy group of population. Hence, the results on stuntedness may have been overestimated. However, the use of the same standard is essential for comparison of the findings since this reference has been used in the past studies [4,10,36] among the indigenous population in Malaysia. Another limitation is the absence of the iron deficiency status of the community studied, a vital aetiology for low hemoglobin concentration and anemia which was not measured in this study. This assessment should be included in future studies.

## Conclusions

Despite government efforts to be inclusive, create equalities and to upgrade the lifestyle through the provision of facilities and amenities among the indigenous, this study highlights a high magnitude of undernutrition and anaemia, and their likely association with STH infections in the present-day hunter-gatherer Negritos after decades of demarginalization. Joint nutritional intervention strategies with mass anti-helminthic treatment are imperative and urgently needed to reduce the undernutrition problems especially among the children. Although the IJV Negritos have some advantages, their undernutrition problem is getting dire as in the RPS due to rapid rate of deforestation and their fate would become similar if no effective measures are taken to arrest the current situation urgently. Nevertheless, demarginalization is definite and inescapable for the present Malaysian indigenous people. Prompt and specific prevention and control strategies that take into account the OA ecology, environment, culture and practices, indigenous knowledge and proactive participation in finding collective solutions should be seriously considered in the overall solution framework. Continued efforts to propagate health education, the establishment of a proper OA health database and monitoring system, and implementation of a good nutrition program are critically and urgently needed for both RPS and IJV communities.

## Supporting information

S1 Fig Study design, summary of sample collection and category of Negrito communities. (PDF)

S1 Table Comparison of anthropometric indices (height and weight) between IJV and RPS Negritos (≤19 years old) according to the age groups (N = 343). (PDF)

S2 Table Demographic, anthropometric and nutritional status among the adult Negritos (>19 y/o) in IJV and RPS communities. (PDF)

S3 Table Hemoglobin (Hb) concentration profiles between the IJV and RPS Negritos (N = 196). (PDF)

S4 Table Potential risk factors associated with stunting (HAZ < -2SD) in the IJV community, (N = 112). (PDF)

S5 Table Potential risk factors associated with stunting (HAZ < -2SD) in the RPS community, (N = 220). (PDF)

S6 Table Potential risk factors associated with wasting (BAZ < -2SD) in the IJV community, (N = 111). (PDF)

S7 Table Potential risk factors associated with wasting (BAZ < -2SD) in the RPS community, (N = 208). (PDF)
