# Supplementary figures and images for: Nutritional status, hemoglobin level and their associations with soil-transmitted helminth infections between Negritos (indigenous) from the inland jungle village and resettlement at town peripheries
Source: PLoS One. 2021 Jan 13;16(1):e0245377. doi: 10.1371/journal.pone.0245377 (PMC7806132; doi:10.1371/journal.pone.0245377)

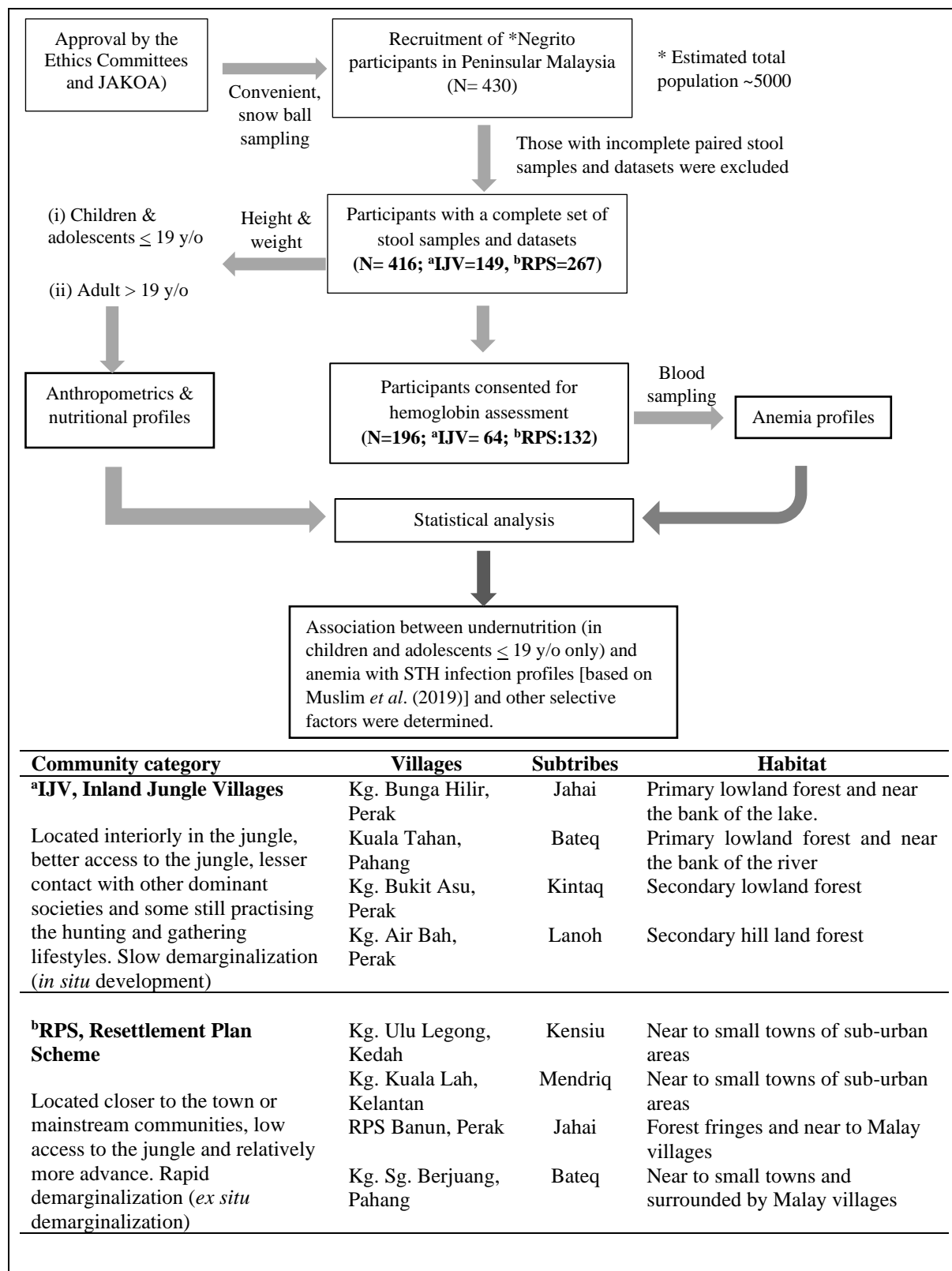

Supplement: S1 Fig — (PDF) [file pone.0245377.s001.pdf]
