# Supplementary material for: Nutritional status, hemoglobin level and their associations with soil-transmitted helminth infections between Negritos (indigenous) from the inland jungle village and resettlement at town peripheries
Source: PLoS One. 2021 Jan 13;16(1):e0245377. doi: 10.1371/journal.pone.0245377 (PMC7806132; doi:10.1371/journal.pone.0245377)
Supplement: S1 Table — (PDF) [file pone.0245377.s002.pdf]

S1 Table: Comparison of anthropometric indices (height and weight) between IJV and RPS Negritos ($\leq$ 19 years old) according to age groups (N=343)

Age range (Years)	Category		Height (cm)	Weight (kg)
		N	**Median (IQR)**	**Median (IQR)**
**2-5**	Overall	71	97.0 (82.0, 105.0)	11.5 (9.8, 14.0)
	IJV	20	98.5 (85.3, 108.8)	12.0 (10.2, 15.0)
	RPS	51	96.0 (81.0, 105.0)	11.5 (9.8, 13.0)
	**P value**		0.21	0.19
**6-8**	Overall	97	112.0 (108.0, 118.0)	16.0 (14.3, 18.0)
	IJV	34	113.0 (108.0, 118.0)	16.0 (13.7, 19.0)
	RPS	63	112.0 (108.0, 118.0)	15.0 (14.5, 17.0)
	**P value**		0.35	0.35
**9-10**	Overall	75	124.0 (118.0, 127.0)	22.0 (19.0-28.0)
	IJV	25	125.0 (122.0, 128.5)	22.0 (20.5, 26.0)
	RPS	50	122.5 (117.0, 127.0)	21.3 (18.0, 28.2)
	**P value**		**0.04***	0.29
**11-19**	Overall	100	141.0 (133.3,146.8)	32.0 (27.0-36.0)
	IJV	38	143.0 (135.0, 148.8)	33.3 (28.9- 38.5)
	RPS	62	135.0 (132.0, 144.5)	29.5 (26.8-34.3)
	**P value**		**0.02***	**0.02***

N= Number of examined participants; P values were calculated based on Mann-Whitney U test to indicate the significant difference between the IJV and RPS communities; IQR= interquartile range
*significant different**, P $\leq$ 0.05**
