# Supplementary material for: Nutritional status, hemoglobin level and their associations with soil-transmitted helminth infections between Negritos (indigenous) from the inland jungle village and resettlement at town peripheries
Source: PLoS One. 2021 Jan 13;16(1):e0245377. doi: 10.1371/journal.pone.0245377 (PMC7806132; doi:10.1371/journal.pone.0245377)
Supplement: S2 Table — (PDF) [file pone.0245377.s003.pdf]

S2 Table: Demographic, anthropometric and nutritional status among the adult Negritos (>19 y/o) in IJV and RPS communities

Variables	Overall (N=73)	IJV (N=32)	RPS (N=41)	P value[b]
	n (%)	n (%)	n (%)	
**Gender**				
Male	27 (37.0)	16 (50.0)	11 (26.8)	0.04*
Female	46 (63.0)	16 (50.0)	30 (73.2)	
**Age**				-
Range	20.0-64.0	20.0- 63.0	20.0 -64.0	
Mean (95 % CI)	30.4 (27.8, 33.0)	28.5 (24.8, 32.3)	31.9 (28.3, 35.5)	
Median (IQR)	29.0 (21.0, 37.0)	24.5 (20.0-33.0)	30.0 (21.5, 39.0)	

Anthropometric	Median (IQR)	Median (IQR)	Median (IQR)	P value[c]
Body weight (kg)	49.0 (46.0, 54.5)	49.3 (46.0, 53.8)	49.0 (46.1, 55.5)	0.91
Body height (cm)	151.0 (147.0, 156.0)	152.5 (147.3, 156.8)	150.0 (147.0, 155.0)	0.38
BMI (kg/m^2)	21.4 (19.6, 25.1)	20.8 (20.7, 24.8)	21.7 (20.7, 24.8)	0.19

Nutritional status[a]	n (%)	95 % CI	n (%)	95 % CI	N (%)	95 % CI	P value[b]
Normal	49 (67.1)	56.3, 77.9	21 (65.6)	49.1, 82.1	28 (68.3)	54.0, 82.6	0.93
Underweight	5 (6.8)	1.0,12.6	2 (6.3)	0.0, 14.7	3 (7.3)	0.0, 15.3	
Overweight & obese	19 (26.0)	15.9, 36.1	9 (28.1)	12.5, 43.7	10 (24.4)	11.3, 37.8	

[a] WHO cut-off reference for nutritional status in adults: Normal (BMI 18.5-24.9); Underweight (BMI <18.5); Overweight & obese (BMI ≥25)

[b] P values were calculated based on Pearson chi-square (χ2) test to indicate the significant difference between the IJV and RPS communities

[c] P values were calculated based on Mann-Whitney U test to indicate the significant difference between the IJV and RPS communities;

* significant different P<0.05
