# Supplementary material for: Nutritional status, hemoglobin level and their associations with soil-transmitted helminth infections between Negritos (indigenous) from the inland jungle village and resettlement at town peripheries
Source: PLoS One. 2021 Jan 13;16(1):e0245377. doi: 10.1371/journal.pone.0245377 (PMC7806132; doi:10.1371/journal.pone.0245377)
Supplement: S3 Table — (PDF) [file pone.0245377.s004.pdf]

S3 Table: Hemoglobin (Hb) concentration profiles among the IJV and RPS communities (N=196)

Variables	Overall (N=196)	IJV (N=64)	RPS (N=132)	
**Hb concentration (g/dL)**	**Mean (95% CI)**	**Mean (95% CI)**	**Mean (95% CI)**	**P value[a]**
**Overall**	10.8 (10.5, 11.0)	10.9 (10.5, 11.2)	10.7 (10.4, 11.0)	0.43
**Gender**				
Male	10.6 (10.3, 11.0)	10.5 (10.1, 10.9)	10.7 (10.2, 11.2)	0.59
Female	10.8 (10.5, 11.2)	11.3 (10.8, 11.9)	10.7 (10.3, 11.1)	0.10
P value (t-test)[b]	0.30	**0.02***	0.68	
**Age groups**				
2-6	9.9 (9.4, 10.5)	10.2 (9.7, 10.8)	9.7 (8.9- 10.5)	0.31
7-12	10.7 (10.4, 10.9)	10.9 (10.4, 11.5)	10.6 (10.2, 10.9)	0.27
≥13	11.7 (11.1, 12.3)	11.4 (10.8, 12.1)	11.9 (11.0, 12.7)	0.42
P value (ANOVA)[c]	**< 0.001***	**0.04***	**< 0.001***	

[a]P value was calculated based on independent t-test between the IJV and RPS; [b]P value was calculated between gender; [c]P value was calculated by ANOVA (Analysis of variance) between age groups; *Significant difference **P ≤ 0.05**
