# Supplementary material for: Nutritional status, hemoglobin level and their associations with soil-transmitted helminth infections between Negritos (indigenous) from the inland jungle village and resettlement at town peripheries
Source: PLoS One. 2021 Jan 13;16(1):e0245377. doi: 10.1371/journal.pone.0245377 (PMC7806132; doi:10.1371/journal.pone.0245377)
Supplement: S4 Table — (PDF) [file pone.0245377.s005.pdf]

S4 Table: Potential risk factors associated with stunting (HAZ < -2SD) in the IJV community, (N=112)

Variables	N	Stunted n (%)	Univariate COR (95% CI)	P value	Multivariate AOR (95% CI)	P value
Male	65	29 (44.6)	1.4 (0.7, 3.1)	0.37	**	**
Female	47	17 (36.2)	1			
Age >10	38	21 (55.3)	2.3 (1.0, 5.1)	0.04*	-	-
Age <u><</u>10	74	26 (35.1)	1			
Family member <u>></u>7	74	31 (41.9)	1.1 (0.5, 2.5)	0.80	**	**
Family member <7	38	15 (39.5)	1			
Income <RM500	70	28 (40.0)	0.9 (0.4, 1.9)	0.77	**	**
Income <u>></u>RM500	42	18 (42.9)	1			
Infected (TT)	83	35 (42.2)	1.2 (0.5, 2.8)	0.69	**	**
Negative	29	11 (37.9)	1			
Moderate-severe TT	56	25 (44.6)	1.3 (0.6, 2.9)	0.44	**	**
Negative-mild	56	21 (37.5)				
Infected (AL)	49	19 (38.8)	0.8 (0.4, 1.8)	0.66	**	**
Negative	63	27 (42.9)				
Moderate-severe AL	30	14 (46.7)	1.4 (0.6, 3.2)	0.52	**	**
Negative-mild	82	32 (39.0)				
Infected (Hkw)	28	14 (50.0)	1.7 (0.7, 3.8)	0.27	**	**
Negative	84	32 (38.1)				
Moderate-severe Hkw	12	7 (58.3)	nc	nc	nc	nc
Negative-mild	100	39 (39.0)				
STH Poly-parasitism	49	22 (44.9)	1.5 (0.7, 3.4)	0.29	**	**
STH Mono-parasitism	52	18 (34.6)				

nc: not computed due to insufficient events per variables of <10;
** No value is available for multivariate analysis because only one factor associated with the stunting in univariate analysis;
*Significant finding of P <u><</u> 0.05
