# Supplementary material for: Nutritional status, hemoglobin level and their associations with soil-transmitted helminth infections between Negritos (indigenous) from the inland jungle village and resettlement at town peripheries
Source: PLoS One. 2021 Jan 13;16(1):e0245377. doi: 10.1371/journal.pone.0245377 (PMC7806132; doi:10.1371/journal.pone.0245377)
Supplement: S5 Table — (PDF) [file pone.0245377.s006.pdf]

S5 Table: Potential risk factors associated with stunting (HAZ < -2SD) in the RPS community, (N=220)

Variables	N	Stunted n (%)	Univariate COR (95% CI)	P value	Multivariate AOR (95% CI)	P value
Female[#]	107	56 (52.3)	0.7 (0.4, 1.2)	0.23	0.7 (0.4, 1.4)	0.32
Male	113	50 (44.2)	1			
Age >10	61	30 (49.2)	1.1 (0.6, 1.9)	0.85	**	**
Age ≤10	159	76 (47.8)	1			
Family member ≥7[#]	151	77 (51.0)	1.4 (0.8, 2.6)	0.22	1.2 (0.6, 2.3)	0.62
Family member <7	69	29 (42.0)	1			
Income ≤RM500[#]	161	71 (44.1)	0.5 (0.3, 1.0)	0.05	0.4 (0.2, 0.8)	0.02*
Income >RM500	59	35 (59.3)	1			
Infected (TT)	157	78 (59.7)	1.2 (0.7, 2.2)	0.48	**	**
Negative	63	28 (44.4)	1			
Moderate-severe TT	108	59 (54.6)	1.7 (1.0, 2.8)	0.60	**	**
Negative-mild	112	47 (42.0)	1			
Infected (AL)	105	52 (49.5)	1.1 (0.7, 1.9)	0.70	**	**
Negative	115	54 (47.0)	1			
Moderate-severe AL	77	39 (50.6)	1.2 (0.7, 2.0)	0.67	**	**
Negative-mild	143	67 (46.9)	1			
Infected (Hkw)[#]	38	23 (60.5)	1.8 (0.9, 3.7)	0.09	1.4 (0.6, 3.2)	0.41
Negative	182	83 (45.6)	1			
Moderate-severe Hkw	9	5 (55.6)	nc	nc	nc	nc
Negative-mild	211	101 (47.9)				
STH Poly-parasitism[#]	101	57 (56.4)	1.9 (1.1, 3.6)	0.02*	1.9 (1.0, 3.4)	0.04*
STH Mono-parasitism	85	32 (39.5)	1			

#Variable included in the logistic multivariate regression analysis because the P value of COR was < 0.25; nc: not computed due to insufficient events per variables of <10;
** No value is available because the respective variable was not included in the multivariate analysis;
*Significant finding of P≤0.05
