# Supplementary material for: Nutritional status, hemoglobin level and their associations with soil-transmitted helminth infections between Negritos (indigenous) from the inland jungle village and resettlement at town peripheries
Source: PLoS One. 2021 Jan 13;16(1):e0245377. doi: 10.1371/journal.pone.0245377 (PMC7806132; doi:10.1371/journal.pone.0245377)
Supplement: S6 Table — (PDF) [file pone.0245377.s007.pdf]

S6 Table: Potential risk factors associated with wasting (BAZ < -2SD) in the IJV community, (N=111)

Variables	N	Wasting n (%)	Univariate COR (95% CI)	P value	Multivariate AOR (95% CI)	P value
Female	63	26 (41.3)	1.5 (0.7, 3.4)	0.28	**	**
Male	48	15 (31.3)				
Age ≤ 10[#]	76	35 (46.1)	4.1 (1.5, 11.0)	0.003*	4.4 (1.6, 11.9)	0.004*
Age > 10	35	6 (17.1)				
Family member ≥7	74	26 (35.1)	0.8 (0.4, 1.8)	0.58	**	**
Family member <7	37	15 (40.5)				
Income ≤RM500[#]	73	32 (43.8)	2.5 (1.0, 6.1)	0.04*	2.7 (1.1, 6.7)	0.03*
Income >RM500	38	9 (23.7)				
Infected (TT)	83	31 (37.3)	1.0 (0.4, 2.6)	0.86	**	**
Negative	28	10 (35.7)				
Moderate-severe TT	56	20 (35.7)	0.9 (0.4, 1.9)	0.79	**	**
Negative-mild	55	21 (38.2)				
Infected (AL)	50	16 (32.0)	0.7 (0.3, 1.5)	0.33	**	**
Negative	61	25 (41.0)				
Moderate-severe AL#	31	8 (25.8)	0.5 (0.2, 1.2)	0.13	0.5 (0.2, 1.2)	0.12
Negative-mild	80	33 (41.3)				
Infected (Hkw)[#]	28	10 (35.7)	0.9 (0.4, 2.3)	0.88	**	**
Negative	83	31 (37.3)				
Moderate-severe Hkw	13	5 (38.5)	1.1 (0.3, 3.5)	0.90	**	**
Negative-mild	98	36 (36.7)				
STH Poly-parasitism	49	17 (34.7)	0.9 (0.4, 1.9)	0.75	**	**
STH Mono-parasitism	53	20 (37.7)				

#Variable included in the logistic multivariate regression analysis because the P value of COR was < 0.25. nc: not computed due to insufficient events per variables of <10;

** No value is available because the respective variable was not included in the multivariate analysis;

*Significant finding of P ≤ 0.05
