# Supplementary material for: Nutritional status, hemoglobin level and their associations with soil-transmitted helminth infections between Negritos (indigenous) from the inland jungle village and resettlement at town peripheries
Source: PLoS One. 2021 Jan 13;16(1):e0245377. doi: 10.1371/journal.pone.0245377 (PMC7806132; doi:10.1371/journal.pone.0245377)
Supplement: S7 Table — (PDF) [file pone.0245377.s008.pdf]

S7 Table: Potential risk factors associated with wasting (BAZ < -2SD) in the RPS community, (N=208)

Variables	N	Wasting n (%)	Univariate COR (95% CI)	P value	Multivariate AOR (95% CI)	P value
Female#<br>Male	109<br>99	58 (53.2)<br>36 (36.4)	2.0 (1.1, 3.5)	0.02*	2.1 (1.1, 3.5)	0.02
Age ≤ 10#<br>Age > 10	152<br>56	76 (50.0)<br>18 (32.1)	2.1 (1.1, 4.0)	0.02*	2.1 (1.1, 4.1)	0.02
Family member ≥7<br>Family member <7	148<br>60	67 (45.3)<br>27 (45.0)	1.0 (0.6, 1.8)	0.97	**	**
Income ≤RM500<br>Income >RM500	155<br>53	70 (45.2)<br>24 (45.3)	1.0 (0.5, 1.9)	0.99	**	**
Infected (TT)<br>Negative	149<br>59	67 (45.6)<br>26 (44.1)	1.1 (0.4, 2.6)	0.88	**	**
Moderate-severe TT<br>Negative-mild	103<br>105	44 (42.7)<br>50 (47.6)	0.8 (0.5, 1.4)	0.48	**	**
Infected (AL)<br>Negative	102<br>106	45 (44.1)<br>49 (46.2)	0.9 (0.4, 1.9)	0.77	**	**
Moderate-severe AL<br>Negative-mild	75<br>133	34 (45.3)<br>60 (45.1)	1.0 (0.6, 1.8)	0.98	**	**
Infected (Hkw)<br>Negative	37<br>171	14 (37.8)<br>80 (46.8)	0.7 (0.3, 1.8)	0.32	**	**
Moderate-severe Hkw<br>Negative-mild	8<br>200	3 (37.5)<br>91 (45.5)	nc	nc	nc	nc
STH Poly-parasitism<br>STH Mono-parasitism	98<br>76	43 (43.9)<br>35 (46.1)	0.9 (0.5, 1.7)	0.78	**	**

#Variable included in the logistic multivariate regression analysis because the P value of COR was <0.25; nc: not computed due to insufficient events per variables of <10;
** No value is available because the respective variable was not included in the multivariate analysis;
*Significant finding of P ≤ 0.05
